# Genome-Wide Development of Polymorphic Microsatellite Markers and Genetic Diversity Analysis for the Halophyte *Suaeda aralocaspica* (Amaranthaceae)

**DOI:** 10.3390/plants12091865

**Published:** 2023-04-30

**Authors:** Wei Xu, Jiancheng Wang, Changyan Tian, Wei Shi, Lei Wang

**Affiliations:** 1 State Key Laboratory of Desert and Oasis Ecology, Xinjiang Institute of Ecology and Geography, Chinese Academy of Sciences, Urumqi 830011, China; xuwei20@mails.ucas.ac.cn (W.X.); www-1256@ms.xjb.ac.cn (J.W.); tianchy@ms.xjb.ac.cn (C.T.); 2 University of Chinese Academy of Sciences, Beijing 100049, China; 3 Turpan Eremophytes Botanical Garden, Chinese Academy of Sciences, Turpan 838008, China

**Keywords:** *Suaeda aralocaspica*, microsatellites, genetic diversity, SSR maker, halophyte

## Abstract

*Suaeda aralocaspica*, which is an annual halophyte, grows in saline deserts in Central Asia with potential use in saline soil reclamation and salt tolerance breeding. Studying its genetic diversity is critical for effective conservation and breeding programs. In this study, we aimed to develop a set of polymorphic microsatellite markers to analyze the genetic diversity of *S. aralocaspica*. We identified 177,805 SSRs from the *S. aralocaspica* genome, with an average length of 19.49 bp, which were present at a density of 393.37 SSR/Mb. Trinucleotide repeats dominated (75.74%) different types of motifs, and the main motif was CAA/TTG (44.25%). We successfully developed 38 SSR markers that exhibited substantial polymorphism, displaying an average of 6.18 alleles with accompanying average polymorphism information content (PIC) value of 0.516. The markers were used to evaluate the genetic diversity of 52 individuals collected from three populations of *S. aralocaspica* in Xinjiang, China. The results showed that the genetic diversity was moderate to high, with a mean expected heterozygosity (He) of 0.614, a mean Shannon’s information index (I) of 1.23, and a mean genetic differentiation index (Fst) of 0.263. The SSR markers developed in this study provide a valuable resource for future genetic studies and breeding programs of *S. aralocaspica*, and even other species in *Suaeda*.

## 1. Introduction

*Suaeda* spp., which has more than 100 species, is a genus of flowering plants that belongs to the family Amaranthaceae [1]. These plants are halophytic herbs or shrubs commonly found in Asia, Europe, North America, and seashores worldwide [2]. *Suaeda* plants are highly adapted to extreme salt and water conditions [3]. They have a unique structure that allows them to store water and tolerate high levels of salinity in the soil [4]. In addition to their medicinal properties, some species of *Suaeda* are used for reclamation of degraded saline lands, as well as in the production of biofuels due to their high oil content [5]. *Suaeda* represents a significant ecological and economic resource for many countries around the world [3].

*Suaeda aralocaspica* is a plant species with succulent leaves that belongs to the family Amaranthaceae. This annual halophyte plant is native to Central Asia and can be found in the salty deserts of the region. In China, this plant is found in the cold desert regions of the Junggar Basin in Xinjiang [6,7]. It carries out complete C_4_ photosynthesis within individual cells but lacks the characteristic leaf anatomy of other C_4_ plants. These features make it potentially valuable in biotechnology of higher photosynthetic efficiencies in agriculturally important C_3_ carbon fixation species such as rice [8]. Seed heteromorphism, which refers to the presence of two distinct types of seeds within a single plant, is a unique characteristic of this species. The two types of seeds are black and brown, and they differ in their size, color, and germination behavior [9,10,11,12]. Conservation measures are imperative for the survival of *S. aralocaspica*, as its population is rapidly diminishing, requiring urgent protection against further decline and potential extinction.

Genetic diversity, also known as gene diversity, is the total range of genetic variation present in different individuals among various populations as well as within the same population [13]. It is considered a fundamental and essential component of biodiversity. Genetic diversity level is critical in determining the long-term survivability and evolutionary capacity of a specific species [14]. A decrease in genetic diversity can lead to a reduction in species’ fitness, ultimately increasing their risk of extinction. For this reason, genetic diversity is often utilized as a predictive tool in studies concerning endangered species and species trend analysis [15,16].

There are several methods for studying genetic diversity, such as morphological tests, cytological markers, biochemical markers, and molecular markers [17,18]. However, molecular markers, in particular, have become one of the most prevalent methods due to their high reliability, independence from environmental factors, and high number [19]. Among molecular markers, microsatellite markers are increasingly popular for conducting genetic studies. Microsatellite markers, also known as simple sequence repeats (SSR) or short tandem repeat (STR) data, a segment of DNA consisting of 1-6bp repeat units in tandem, offer several advantages, including high information content, co-dominance, and multiple alleles. Therefore, they are widely used in various genetic studies as an effective molecular marker [20,21].

There have been limited research efforts focused on exploring the genetic diversity of *Suaeda* species [22,23]. This could be attributed to the fact that molecular markers specific to the *Suaeda* genus have not been extensively developed. Genetic diversity of *S. corniculata* from Bunge in Eastern Siberia was analyzed by using five inter-simple sequence repeats (ISSRs) markers [24]. In a study conducted by Prinz (2013), the genetic diversity and differentiation of 31 populations of *S. maritima* from coastal and inland areas of Central Europe were compared using 10 polymorphic microsatellite markers. The findings revealed that there were significant differences in the genetic diversity between populations of *S. maritima* from coastal and inland areas. This suggests that the addition of anthropogenic salt sites may have a facilitative effect on gene flow in inland populations of saline plants [25]. Prinz (2009) also developed a set of 12 polymorphic microsatellite markers specifically designed for analyzing the genetic diversity of *S. maritima*. These polymorphic markers were also found to be cross-amplifiable across related species, such as *S. glauca* and *S. salsa*. Of the 12 markers, 11 were shown to be reproducible and effective for conducting genetic analyses of *Suaeda* populations [8]. The limited research on genetic diversity in *Suaeda* species suggests moderate to high levels of genetic diversity within populations and variable levels of diversity among populations. The genome of *S. aralocaspica*, comprising 452 Mb, has been sequenced to provide a data base for the analysis and development of SSR markers for *S. aralocaspica* and even the genus *Suaeda* [26].

The habitat of *S. aralocaspica* is facing significant fragmentation and degradation caused by human activities and climate change in China. This fragmentation is posing a potential threat to the species as it may reduce gene flow among isolated populations. The primary aim of this research is to conduct a comprehensive genome-wide study to characterize and develop SSR markers in *S. aralocaspica*. This study also aims to analyze the genetic diversity of SSRs in *S. aralocaspica* populations to understand the species’ population structures. The developed SSR markers can be used to identify genetic variations and patterns in different populations of *S. aralocaspica*, which may be useful in conservation planning and germplasm management in the future.

## 2. Results

### 2.1. Analysis of the Distribution of SSRs in the Genome of S. aralocaspica

MISA software was utilized to screen 452 Mb of the entire genome of *S. aralocaspica*, which led to the detection of 177,805 SSR markers. The total length of these markers amounted to 3,465,418 bp. The frequency and density of SSR in the entire genome were determined to be 393.37SSR/Mb and 7666.85 bp/Mb, respectively, representing approximately 0.77% of the whole genome sequence (Table 1).

**Figure 1:**
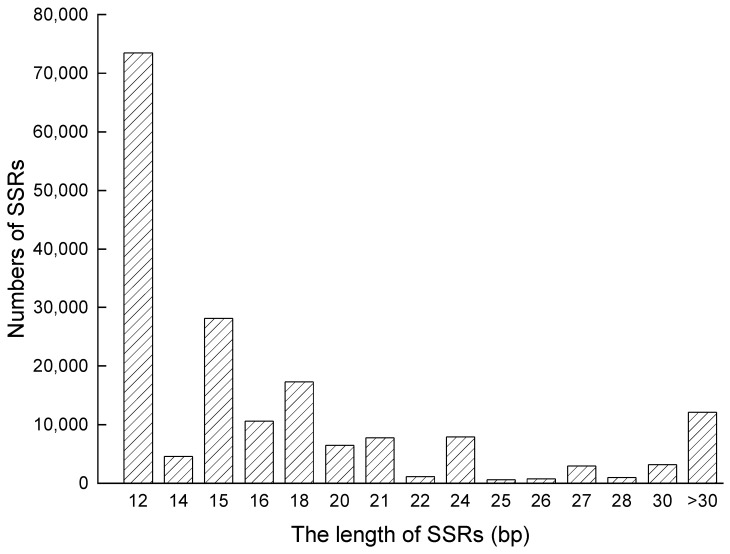
Length distribution of SSRs in the *S. aralocaspica* genome.

**Figure 2:**
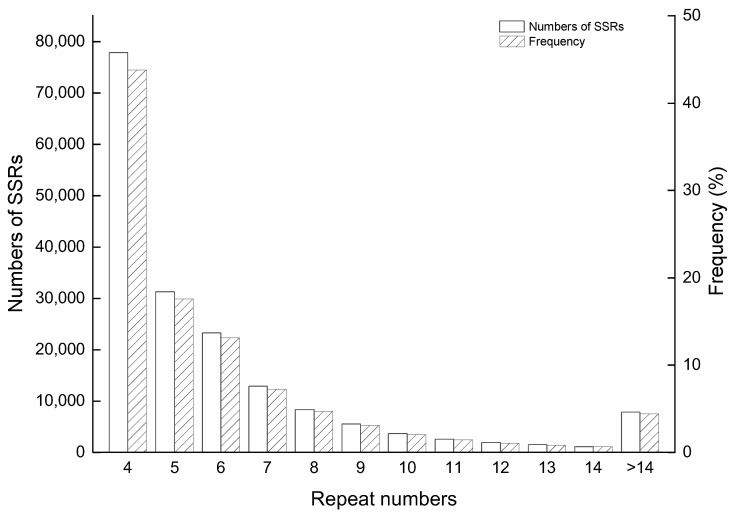
Distribution of repeat numbers of SSRs in the *S. aralocaspica* genome.

The length of SSRs found on the complete genome of *S. aralocaspica* was from 12 bp to 9862 bp, with an average length of 19.49 bp. The most frequently occurring repeat length was 12 bp, which appeared 73,445 times, and was followed by 15 bp, 18 bp, and 16 bp, occurring at frequencies of 28,116, 17,288, and 10,626, respectively (Figure 1). An analysis of the repeated motifs at each SSR locus revealed that the number of repeats ranged from 4 to 1551, and the majority of loci had four tandem repeats (43.77%), followed by those with five tandem repeats (17.57%) (Figure 2).

Trinucleotide repeats were the most common, followed by dinucleotide, tetranucleotide, pentanucleotide, and hexanucleotide repeats in the whole genome of *S. aralocaspica*. The total length of SSRs in the genome was found to be 3,465,418 bp, and the total length of SSRs with di-, tri-, tetra-, penta-, and hexanucleotide repeats was 473,878 bp, 2,383,446 bp, 337,516 bp, 113,540 bp, and 157,038 bp, respectively. The average length of each basic sequence was 18.76 bp, 17.70 bp, 30.27 bp, 30.11 bp, and 52.96 bp, respectively (Table 2).

In the entire genome of *S. aralocaspica*, a total of 177,805 SSRs were identified, which contained 355 nucleotide repeats. These repeats were categorized into di-, tri-, tetra-, penta-, and hexanucleotide repeats, with 4, 10, 32, 88, and 221 nucleotide repeats, respectively. The most commonly used repeat motif was AAC/GTT (44.25%), followed by AT/AT (10.08%), AAAT/ATTT (2.85%), AAAAT/ATTTT (0.54%), and AACAAT/ATTGTT (0.15%). Among all the repeat motifs, di-, tri-, and tetranucleotide repeats were found to be the most prevalent types, while the proportion of five- and six-nucleotide repeats was considerably smaller, accounting for only 3.79% of the total 309 nucleotide repeats (Table 3).

### 2.2. Development of Genome SSR Markers for S. aralocaspica

A set of 100 candidate SSR pairs was chosen for *S. aralocaspica*, out of which, 88 were successful in producing clear bands with eight or more SSR loci. These loci were further tested for polymorphism using UV observation of PCR products and 2% agar gel electrophoresis. The resulting data was analyzed using Cervus v3.0.7, which identified 38 highly polymorphic SSR loci (Table S1). These loci were then used to amplify three distinct populations of *S. aralocaspica*.

A total of 52 samples were collected from three populations of *S. aralocaspica* and were analyzed using 38 SSR loci (Table 4). The results indicated that these loci contained a total of 235 alleles, with an average of 6.18 Na (number of alleles) per individual locus, ranging between 3 and 12. Locus SA-di-63 and SA-te-94 had the highest number of alleles, while SA-te-29 and SA-te-98 had the lowest. The average value of Ne (effective alleles) across all loci was 2.96, with a maximum of 5.131 observed at locus SA-tri-85 and a minimum of 1.405 at locus SA-di-65. The range of variation for I was 0.596 to 1.827, with the highest value observed at locus SA-tri-85 and the lowest at SA-di-65. The Ho values ranged from 0 to 1, while the He values ranged from 0.288 to 0.805. The uHe values ranged from 0.291 to 0.813, and the Fst values ranged from 0.027 to 0.548, with an average value of 0.263, indicating a significant genetic divergence among the collected *S. aralocaspica* samples. The range of PIC values was 0.272 to 0.773, with a mean value of 0.568. The majority of the loci had PIC values above 0.5, indicating good polymorphism in the 38 primer pairs that were screened.

### 2.3. Genetic Diversity Analysis of S. aralocaspica Populations

The Na values of the selected markers amplified for the three populations of *S. aralocaspica* ranged from 2.921 to 4.447, while Ne ranged from 1.794 to 2.667. The values for I ranged from 0.642 to 1.085, Ho ranged from 0.207 to 0.299, He ranged from 0.365 to 0.573, uHe ranged from 0.376 to 0.591, and F ranged between 0.515 and 0.602 (Table 5).

The analysis of molecular variation (AMOVA) in the *S. aralocaspica* population revealed that 35% of the genetic variation was present within individuals, 32% varied between populations, and 33% was seen between individuals.

**Figure 3:**
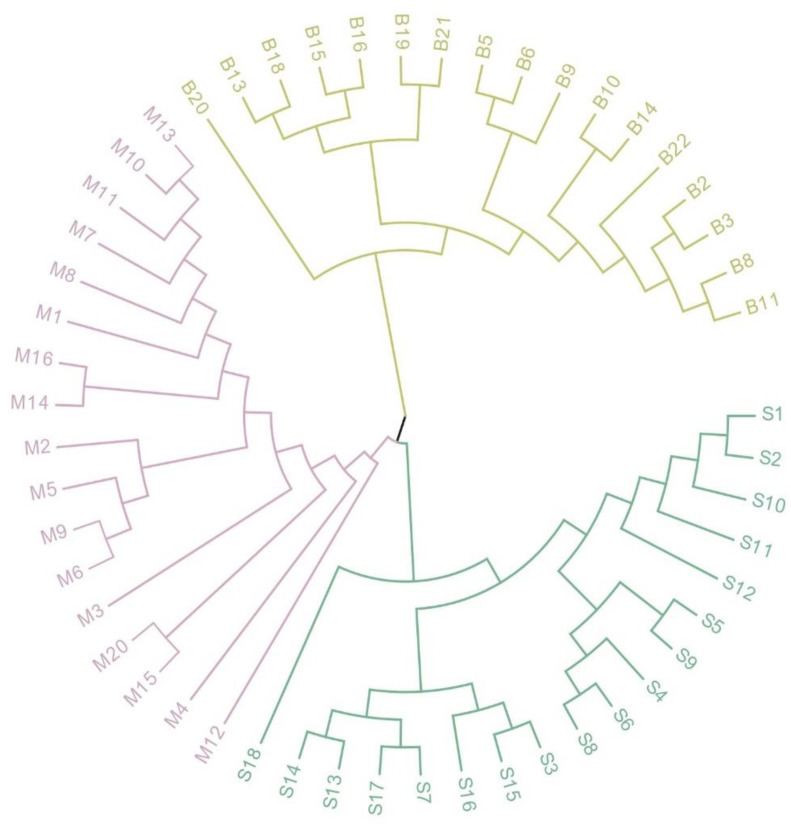
Unweighted pair group method with arithmetic mean (UPGMA) tree based on Nei’genetic distances calculated for *S. aralocaspica*. UPGMA circular dendrograms based on Nei’s genetic distances using Mega 6, with different colors representing different populations, population Shawan (S1–S18) in green, population Shihezi (M1–M16, M20) in pink, and population Fukang (B2–B22, missing B4, B7, B12, B17) in yellow.

In the populations studied, the genetic identity between the Shawan and Shihezi *S. aralocaspica* populations was found to be the highest, at 0.654. On the other hand, the genetic identity between Fukang-Shawan and Fukang-Shihezi pairs was lower, at 0.438 and 0.548, respectively (Table 6). This was further supported by the UPGMA analysis, where the Shawan and Shihezi populations were found to be genetically distant from each other, while the Fuakang and Shawan populations showed the most differences in genetic identity (Figure 3). 

**Figure 4:**
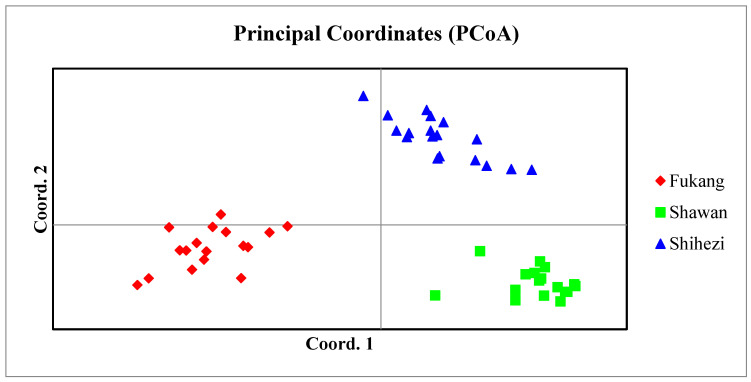
Principal coordinate analysis of three groups of *S. aralocaspica* based on SSR genetic distance. The different colors and shapes represent the different populations: red diamonds for the Fukang population, green squares for the Shawan population, and blue triangles for the Shihezi population. The axis labels list the percentage of explained variance for each principal coordinate.

The analysis using PCoA indicated that the initial principal coordinate encompassed 31.91% of the overall genetic variation, with the second and third principal coordinates accounting for 14.72% and 12.60%, respectively. The combined eigen-values for the three principal coordinates reached 59.22% (Figure 4).

## 3. Discussion

*Suaeda* is a valuable representative plant species among saline plants, with significant research potential. However, the development of molecular markers for *Suaeda* has been limited, hindering the progress of molecular ecology and population genetics studies on this genus. To address this gap, we utilized the sequencing data of *S. aralocaspica* to identify SSRs on its genome and subsequently developed 38 SSR markers with good polymorphism. These markers can be used to analyze the genetic diversity of *S. aralocaspica* and contribute to the study of genetic diversity and structure of the *Suaeda* plants.

After performing the analysis, we discovered that the frequency of SSRs in *S. aralocaspica*’s entire genome was 393.37 SSRs/Mb. This amount was significantly higher when compared to *Anemone coronaria* (65.52 SSRs/Mb), *Solanum melongena* (120 SSRs/Mb), and *Triticum aestivum* (36.68 SSRs/Mb) [27,28,29]. Therefore, it can be inferred that SSRs were abundant in the whole genome of *S. aralocaspica*. Dinucleotide and trinucleotide repeats were the most common SSRs in the genome of *S. aralocaspica*, similar to Tartary buckwheat [30]. However, there is a difference in abundance between the two, as dinucleotide repeats were the most common in Tartary buckwheat (63.95%), whereas trinucleotide repeats were more common in *S. aralocaspica* (75.74%).

In this study, we examined the genetic diversity of *S. aralocaspica* using 38 pairs of SSR primers developed for various populations. We found that the expected heterozygosity (He) and Shannon’s information index (I) values for the three populations ranged from 0.288 to 0.805 and 0.642 to 1.085, respectively. A comparison of our results with those obtained for other species, such as *S. maritima* (He = 0.37, I = 0.97) [25], *S. corniculata* subsp. *mongolica* (I = 0.1688), *S. “jacutica”* (I = 0.0878), and *S. corniculata* s. str [23]. *Nuphar submersa* (He = 0.42), *Pedicularis kansuensis* (He = 0.441, I = 0.781), *Ruta oreojasme* (He = 0.687), *Vincetoxicum atratum* (He = 0.67), *Ammi seubertianum* (He = 0.66, I = 1.28), *Ammi trifoliatum* (He = 0.67, I = 1.35), and *Tapiscia sinensis* (He = 0.6904, I = 1.4368), revealed some variation in values [31,32,33,34,35,36]. It is essential to note that different plants and SSR loci, as well as the number of markers used, can all affect genetic diversity analysis results.

The genetic diversity analysis of *S. aralocaspica* populations from three distinct regions revealed differences in their genetic diversity. Specifically, the genetic diversity of *S. aralocaspica* populations in Fukang was significantly greater than that of populations in Shihezi and Shawan. This may be due to the different habitats of the three populations. For instance, the *S. aralocaspica* population in Fukang is located near a protected reservoir, making it less susceptible to human activities and boasting relatively stable conditions. It also has a larger habitat area and population size compared to those in Shihezi and Shawan. Conversely, the *S. aralocaspica* populations in Shihezi and Shawan are more frequently impacted by human activities and experience greater environmental volatility. This may explain the significant differences in genetic diversity observed between the three populations.

In this study, we compared the genetic identity and actual geographic distance of three distinct *S. aralocaspica* populations. Our findings revealed that the *S. aralocaspica* populations of Shihezi and Shawan had the closest geographic distance and the highest genetic identity. Conversely, the Fukang and Shawan populations showed the greatest geographic distance and the lowest genetic similarity. These results indicate an inverse relationship between genetic identity and geographic distance among the three *S. aralocaspica* populations. This suggests that geographic distance may play a vital role in influencing gene flow among different *S. aralocaspica* populations.

Gene flow has a reducing effect on genetic differentiation between populations, particularly where gene flow is greater than 1 number of migrants (Nm). However, when Nm is less than 1, local differentiation between populations tends to occur. Despite this, the collected samples of *S. aralocaspica* indicate greater genetic differentiation, with mean Fst values above 0.25. This suggests that *S. aralocaspica* may have undergone local adaptation due to high selection pressure, which can occur even in the presence of high levels of gene flow, according to Endler et al. [37]. In combination with other data, it is possible that high selection pressure has contributed to the local adaptation of *S. aralocaspica* [38]. Factors that influence plant genetic diversity include species-related factors such as mating systems, bottleneck effects, evolution, and life history, as well as anthropogenic factors.

Heterozygous species typically exhibit greater genetic diversity than self-incompatible species [39,40]. Although there is no definitive evidence on the mating system of *S. aralocaspica*, its monoecious annual nature and inbreeding coefficients exceeding 0.5 in all three populations suggest a likelihood of more pronounced inbreeding. Further analysis is necessary to determine the exact mating system. Mating among close relatives in small populations often happens out of necessity and results in high inbreeding coefficients and a decline in genetic diversity [41]. *S. aralocaspica* populations exhibit high genetic diversity and this may be attributed to natural or anthropogenic factors that have caused severe habitat fragmentation and a significant reduction in population size, with the small populations inheriting a fraction of the genetic variation from the original large populations. Further investigation is needed to establish the exact causes of this phenomenon.

## 4. Materials and Methods

### 4.1. Plant Material

The 52 fresh plant samples utilized were obtained from three distinct populations of *S. aralocaspica* (17 individuals from Fukang (87°40′ E, 44°13′ N), 17 individuals from Shihezi (87°14′ E, 44°45′ N), and 18 individuals from Shawan (85°50′ E, 44°36′ N)) in July 2021 from Xinjiang, China. Fresh leaves of *S. aralocaspica* within different habitats were collected using the quadrat method. After collection, the samples were carefully wrapped in tinfoil and preserved in liquid nitrogen to maintain their freshness. Subsequently, the plant materials were stored in an ultra-low-temperature refrigerator at −80 °C until they were utilized for subsequent experiments.

### 4.2. Genome SSR Identification and Development

This study utilized whole genome sequencing results of *S. aralocaspica*, obtained from the open access data of NCBI (with project registration number PRJNA428881), as a basis for developing a set of SSR primers with high polymorphism. In order to achieve this, our study considered microsatellite markers with a standard size of 2–6 bp, excluding single nucleotides. To determine the microsatellite loci, the MISA software was utilized, focusing on nucleotide microsatellites with a minimum repeat number of 4. We then used Primer 3 software to design primers specific to flanking genomic sequences based on the read parameters of the microsatellite regions. The expected amplicon length of our design ranged from 100 to 300 bp.

### 4.3. PCR Amplification, and Electrophoresis Detection 

Genomic DNA from *S. aralocaspica* plant material was extracted using the DNAsecure Plant Kit (Tiangen Biotechnology, Beijing, China), following the manufacturer’s guidelines. From all genomic SSRs, 100 candidate primer pairs were randomly selected based on the minimum lengths of 14, 18, and 20 bp for di-, tri-, and tetranucleotide repeats [42,43], respectively. The forward primers’ 5′ ends were labeled with FAM blue, a fluorescent dye (Shanghai General Biotechnology, Shanghai, China), for easy scoring in genotyping. PCR amplification was performed using the selected primers for all sample DNA in a 25 μL reaction system containing 1 μL of template DNA, 1 μL each of upstream and downstream primers, 2 × EasyTaq PCR SuperMix 12.5 μL, and ddH_2_O 9.5 μL. The amplification reaction procedure consisted of three stages: pre-denaturation at 94 °C for 3 min in the first stage and, in the second stage, 30 cycles of denaturation at 94 °C for 30 s, annealing at 58 °C for 30 s, and extension at 72 °C for 30 s. Successful amplification was confirmed by analyzing at least eight or more of the ten individuals’ bright bands using 2% agarose gel electrophoresis. Alleles (.FSA) generated after the PCR amplification experiment were analyzed using GeneMarker v2.2.0 (SoftGenetics LLC., State College, PA, USA) genotyping software. Primers with good polymorphism were selected for amplification of the collected *S. aralocaspica* population material by data analysis, followed by analysis of the amplification results.

### 4.4. Data Analysis

Various genetic diversity indices were computed using different software packages. Popgene 32 [44], Cervus v3.0.7, and GenAlEx 6.5 were used to calculate the number of alleles (Na), number of effective alleles (Ne), Shannon information index (I), observed heterozygosity (Ho), expected heterozygosity (He), and polymorphism information content (PIC) [45]. The Nei’s gene diversity index was also calculated using the Nei’s genetic distance. Subsequently, UPGMA clustering was performed for all *S. aralocaspica* samples using Mega6 [46]. Principal coordinate analysis and genetic similarity analysis were carried out using GenAlEx 6.5 software. DataFormater 2010 was used to convert the data types [47].

## 5. Conclusions

Based on the sequencing of the whole genome of *S. aralocaspica*, this study has successfully developed a large set of polymorphic microsatellite markers that can be used for the genetic diversity analysis of halophyte *S. aralocaspica*. The results of the genetic diversity analysis showed that the populations of *S. aralocaspica* had moderate levels of genetic differentiation and high levels of genetic diversity within each population. The microsatellite markers developed in this study provide a valuable tool for future population genetics studies and conservation efforts, not just for this species but also for other *Suaeda* species. Overall, this study highlights the importance of genetic diversity analysis in understanding the adaptive potential and conservation of halophyte species in changing environments.

## Figures and Tables

**Table 1:** Overview of SSRs in the *S. aralocaspica* genome.

Items	Numbers
Total size of genome (Mb)	452
Total number of identified SSRs	177,805
Total length of SSRs (bp)	3,465,418
Frequency (SSRs/Mb)	393.37
Density (bp/Mb)	7666.85
Total content of genome SSRs(%)	0.77

**Table 2:** SSR markers analysis of *S. aralocaspica*.

Repeat Type	Predominant Type	Number	Proportion (%)	Frequency (SSRs/Mb)	Total Length (bp)	Average Length (bp)
Di	AT	25,256	14.20	55.88	473,878	18.76
Tri	AAC/GTT	134,663	75.74	297.93	2,383,446	17.70
Tetra	AAAT/ATTT	11,150	6.27	24.67	337,516	30.27
Penta	AAAAT/ATTTT	3771	2.12	8.34	113,540	30.11
Hexa	AACAAT/ATTGTT	2965	1.67	6.56	157,038	52.96
Total		177,805	100	393.37	3,465,418	19.49

**Table 3:** Main motif of *S. aralocaspica* genome SSRs.

The Motif of Repeat	Repeat Numbers						Total	Percentage (%)
	4	5	6	7	8	>8		
AT/AT	0	0	4595	3065	2511	7751	17,922	10.08
AG/CT	0	0	1594	913	549	1359	4415	2.48
AC/GT	0	0	1110	625	401	748	2884	1.62
AAC/GTT	34,600	18,327	10,383	5476	3172	6728	78,686	44.25
AAT/ATT	10,890	3893	2108	1243	904	5491	24,529	13.80
ACC/GGT	5994	2217	966	438	248	155	10,018	5.63
AAG/CTT	6119	1338	439	165	70	317	8448	4.75
ATC/ATG	4297	1245	481	219	107	207	6556	3.69
ACT/AGT	1229	481	273	130	73	237	2423	1.36
AGC/CTG	1429	277	76	30	9	20	1841	1.04
AGG/CCT	1068	262	95	47	31	47	1550	0.87
AAAT/ATTT	2861	1037	457	220	111	375	5061	2.85
AATC/ATTG	962	142	34	8	4	4	1154	0.65
AATT/AATT	562	151	47	28	2	4	794	0.45
AAAAT/ATTTT	709	182	38	10	7	6	952	0.54
Total	70,720	29,552	22,696	12,617	8199	23,449	167,233	94.05
Percentage (%)	39.77	16.62	12.76	7.10	4.61	13.19	94.05	

**Table 4:** Genetic diversity parameters of 38 polymorphic SSR loci developed for *S. aralocaspica*.

Locus	N	Na	Ne	I	Ho	He	uHe	Fst	PIC
SA-di-11	52	4	2.116	1.004	0.000	0.527	0.532	0.243	0.488
SA-di-13	52	4	2.392	1.007	0.808	0.582	0.588	0.100	0.517
SA-tri-24	52	7	4.989	1.755	0.038	0.800	0.807	0.390	0.773
SA-tri-26	52	6	3.462	1.354	0.385	0.711	0.718	0.287	0.658
SA-te-29	52	3	1.702	0.730	0.173	0.413	0.417	0.261	0.369
SA-di-34	50	4	2.417	1.057	0.060	0.586	0.592	0.158	0.526
SA-di-35	52	6	2.973	1.294	0.019	0.664	0.670	0.548	0.609
SA-di-39	52	5	2.818	1.276	0.038	0.645	0.651	0.278	0.605
SA-di-40	52	3	2.000	0.791	0.038	0.500	0.505	0.198	0.408
SA-tri-41	52	6	3.485	1.400	0.231	0.713	0.720	0.096	0.664
SA-tri-42	52	4	2.094	0.942	0.019	0.522	0.527	0.342	0.469
SA-tri-43	51	4	1.676	0.689	0.020	0.403	0.407	0.152	0.343
SA-tri-46	52	8	3.634	1.553	0.212	0.725	0.732	0.314	0.69
SA-te-50	52	8	2.917	1.331	0.212	0.657	0.664	0.258	0.597
SA-di-53	52	10	4.650	1.809	0.154	0.785	0.793	0.415	0.757
SA-di-54	52	5	2.528	1.093	0.038	0.604	0.610	0.407	0.528
SA-di-59	52	5	2.367	1.063	0.077	0.577	0.583	0.528	0.516
SA-di-61	51	9	3.315	1.433	0.961	0.698	0.705	0.127	0.648
SA-di-62	50	7	2.244	1.177	0.080	0.554	0.560	0.456	0.524
SA-di-63	51	12	3.605	1.655	0.412	0.723	0.730	0.209	0.69
SA-di-64	52	6	5.017	1.685	0.692	0.801	0.808	0.129	0.771
SA-di-65	52	4	1.405	0.596	0.135	0.288	0.291	0.160	0.272
SA-di-67	52	8	4.404	1.674	0.000	0.773	0.780	0.469	0.74
SA-di-69	52	8	4.265	1.697	1.000	0.766	0.773	0.027	0.734
SA-di-73	52	6	2.041	1.109	0.058	0.510	0.515	0.258	0.487
SA-di-74	52	6	1.992	1.014	0.038	0.498	0.503	0.338	0.464
SA-tri-77	52	6	3.453	1.373	1.000	0.710	0.717	0.096	0.664
SA-tri-78	51	7	2.876	1.419	0.078	0.652	0.659	0.350	0.623
SA-tri-83	52	7	4.765	1.649	0.019	0.790	0.798	0.265	0.757
SA-tri-85	52	10	5.131	1.827	0.135	0.805	0.813	0.398	0.778
SA-tri-86	52	9	4.122	1.671	0.173	0.757	0.765	0.297	0.727
SA-tri-88	52	4	2.687	1.122	0.981	0.628	0.634	0.063	0.559
SA-te-92	52	6	2.098	0.964	0.654	0.523	0.528	0.162	0.456
SA-te-93	52	5	2.155	0.904	0.173	0.536	0.541	0.350	0.444
SA-te-94	52	12	3.532	1.702	0.077	0.717	0.724	0.306	0.691
SA-te-96	52	4	1.528	0.691	0.019	0.346	0.349	0.105	0.323
SA-te-97	52	4	1.648	0.756	0.058	0.393	0.397	0.337	0.359
SA-te-98	51	3	1.922	0.711	0.020	0.480	0.484	0.128	0.374

Note: N = number of individuals sampled; Na = number of alleles; Ne = effective alleles I = Shannon information index; Ho = observed heterozygosity; He = expected heterozygosity; uHe = unbiased expected heterozygosity; PIC = polymorphism information content; Fst = fixation index.

**Table 5:** Population genetic characteristics based on 38 SSR loci data in three populations of *S. aralocaspica*.

Pop		N	Na	Ne	I	Ho	He	uHe	F
Fukang	Mean	16.895	4.447	2.667	1.085	0.299	0.573	0.591	0.515
	SE	0.063	0.269	0.155	0.062	0.053	0.027	0.028	0.078
Shawan	Mean	16.974	2.921	1.794	0.642	0.207	0.365	0.376	0.602
	SE	0.026	0.265	0.113	0.066	0.061	0.035	0.036	0.109
Shihezi	Mean	17.895	2.947	1.920	0.712	0.228	0.416	0.428	0.571
	SE	0.063	0.196	0.114	0.056	0.058	0.031	0.032	0.098

Note: N = number of individuals sampled; Na = number of alleles; Ne = effective alleles I = Shannon information index; Ho = observed heterozygosity; He = expected heterozygosity; uHe = unbiased expected heterozygosity; F= inbreeding factor.

**Table 6:** Nei’s unbiased measures of genetic identity for three populations of *S. aralocaspica*.

	Fukang	Shawan	Shihezi
Fukang	1		
Shawan	0.438	1	
Shihezi	0.548	0.654	1

## Data Availability

The data presented in this study are available on request from the corresponding author.

## Supplementary Materials

The following supporting information can be downloaded at: https://www.mdpi.com/article/10.3390/plants12091865/s1, Table S1. Characteristics of the 38 polymorphic SSR primer pairs of *S. aralocaspica*. (supplementary material).
