# Supplementary material for: Genome-Wide Development of Polymorphic Microsatellite Markers and Genetic Diversity Analysis for the Halophyte Suaeda aralocaspica (Amaranthaceae)
Source: Plants (Basel). 2023 Apr 30;12(9):1865. doi: 10.3390/plants12091865 (PMC10181123; doi:10.3390/plants12091865)
Supplement: Supplementary file 1 [file plants-12-01865-s001.zip › plants-2324492-supplementary.pdf]

Table S1. Characteristics of the 38 polymorphic SSR primer pairs of *S. aralocaspica*.(supplementary material)

Loci	Primer sequences (5′–3′)	Repeat motif	Fluorescent dyeb	Length_of_PCR_produces
SA-di-11	F: ATGTTTCTTGGCATGACTCAAAT<br>R: ATCTCCTGGAGTATCCCAGCTAC	$(GA)_8$	5'-FAM	139
SA-di-13	F: GAGAAGTGATATTGGTGGGAGTG<br>R: ACCAAACATTTAACTCGTCCCTC	$(AG)_8$	5'-FAM	152
SA-tri-24	F: ATGGTCCGACGTATGACAATACT<br>R: CGACAATGAAGATGGTAATGAGA	$(ATT)_7$	5'-FAM	126
SA-tri-26	F: TAGTAAGAAGGAGGAGGAGGAGG<br>R: AGATTGTTTCGACACTAGCAAGC	$(AAG)_7$	5'-FAM	140
SA-te-29	F: CATTGGCTTGAGCTTCACTTACT<br>R: CTTGGTCACACCTTCCTCAATA	$(AAAG)_6$	5'-FAM	134
SA-di-34	F: ACAGAAGATGAAGCAGTGAAAGG<br>R: ATTTTCTTTGTCTTTTGGCCTTC	$(GA)_8$	5'-FAM	111
SA-di-35	F: GAAAGGTACGTGATGACACATAGG<br>R: GGGAAATAAAAGGTTGCATGTACT	$(AT)_9$	5'-FAM	160
SA-di-39	F: CCTTATTAGCTTCCATTGTTTCAA<br>R: TGTATGCCCATATTGTTAGACGTT	$(TA)_9$	5'-FAM	125
SA-di-40	F: TATAAATACTCCGACCTGCAACG<br>R: TAAAGTTTGCGAAGATGATCGAC	$(CT)_7$	5'-FAM	158
SA-tri-41	F: AACAACAAACAGACCTTATTCGG<br>R: TTGGACCATCATCATTAGTGACA	$(ATG)_6$	5'-FAM	156
SA-tri-42	F: TCAAAAGTTTCTCTCAATTCATGG<br>R: AGATGCAGACTAACCAGCAAAAG	$(TAA)_6$	5'-FAM	139
SA-tri-43	F: CCCTGCTGTAATTGTTTGATTTAC<br>R: ACTCCTTTTCGCCAATAATAAAA	$(ATT)_7$	5'-FAM	160
SA-tri-46	F: TGCTAGAAGATTTATATTGTCAAACG<br>R: AAATTGAACTGAAGTAACCGTAAAAA	$(ATA)_7$	5'-FAM	158
SA-te-50	F: CTCAACACATTTCTCACTCTCCC<br>R: TACTTGAAACAAATCCCCTCAGA	$(TTTC)_6$	5'-FAM	154
SA-di-53	F: GCCTTTTTCCCTTTACCTACCTA<br>R: GCCACTCATGGTTATTATTTTATTTG	$(TA)_{11}$	5'-FAM	198
SA-di-54	F: ACCTTCACATTCACCTTCAAAAA<br>R: ACCAAGGTTTTCATCCTCATCTT	$(AC)_{15}$	5'-FAM	142
SA-di-59	F: TTCTTGCATAATCACTCTCCCTC<br>R: TATGAAGATGCTCCCTGCACTAT	$(CT)_{11}$	5'-FAM	132
SA-di-61	F: GGAAAATGAGAACATTCTAACATCAA<br>R: TCCAACCACTTCCAAAAGTAGAA	$(AT)_7$	5'-FAM	148
SA-di-62	F: TGAAAAGGAAATAGTGACAAGCC<br>R: TTTTTGTGGTTTTCATGGTGA	$(AT)_{13}$	5'-FAM	125
SA-di-63	F: CTTTGCCCTTCATTCATCACTT	$(CT)_8$	5'-FAM	156

	R: GTAATTAGTGTTTTGCGGAATGC			
SA-di-64	F: TGCACAATAAAACACGTAACTGG<br>R: GCAAAACTTTCTTGCACAAAGAC	$(AG)_7$	5'-FAM	111
SA-di-65	F: CAATGAGTATGCGGATTTGACTT<br>R: CCGCTTTAAGTCTTATATGTACCCA	$(TA)_7$	5'-FAM	160
SA-di-67	F: AAAATAGAACGACTTCCAGGTCC<br>R: GTGTTTACTCGTGACAGGGTCTC	$(AC)_7$	5'-FAM	150
SA-di-69	F: GAGAGAAAGGAAGAGAAAGGCAC<br>R: ATTCGATCCTCAACTCACACATT	$(AG)_{10}$	5'-FAM	121
SA-di-73	F: TATTTACGTGCATGCTGTATTGG<br>R: GATGGTGTACCGAATTCTGATGT	$(TG)_{12}$	5'-FAM	145
SA-di-74	F: TTACCATATGCATGTTTGTCTTGA<br>R: CAATTGGTACCTAAATTCACCTCC	$(CT)_{11}$	5'-FAM	154
SA-tri-77	F: AGTAGGGGACTCCTCTCAGTCAG<br>R: CGTCATGGTATCACTTCAACCTT	$(CAT)_7$	5'-FAM	152
SA-tri-78	F: TATATGAAATTGCTGCTTCGTGA<br>R: TGAGAATGACACTGAAAAAGATGA	$(TTA)_8$	5'-FAM	142
SA-tri-83	F: AAGGCACAAGCAAAATGAAATAA<br>R: TCGAGTACACATTTTTGGGATTT	$(AAT)_6$	5'-FAM	155
SA-tri-85	F: TATCCCAACGCCTCTTTTATTTT<br>R: GCTGCGTTTGATTTTTATTTTTG	$(AAT)_{11}$	5'-FAM	125
SA-tri-86	F: TGTATGCTATTGCTTGCCTAAGA<br>R: GACCAAAGGTCGCTATTTTATGA	$(GTT)_9$	5'-FAM	204
SA-tri-88	F: TCGAATCAAACTGAAATTAAGCTG<br>R: ATTGCCGGGATTATTATTAGCAT	$(AAT)_9$	5'-FAM	159
SA-te-92	F: GGGTTTATCCTTGTCCTTGTGTAA<br>R: GACAAATGAGTCCACAAGTAGCC	$(TATT)_5$	5'-FAM	158
SA-te-93	F: GGAAAAAGCTAGTCAATGTCCAA<br>R: TTAATGTGATGAATCTGAATGCG	$(AAAT)_5$	5'-FAM	153
SA-te-94	F: TGATTATCAAATCAACTCCCCAC<br>R: CAAAGAAATATTGAAATTCCTCAACA	$(TTTA)_7$	5'-FAM	155
SA-te-96	F: TGCCACAGGTATGCATCTTTAAT<br>R: TCTGAAAGGCAAACCAACATTAT	$(ATGT)_5$	5'-FAM	158
SA-te-97	F: TGGTCGACTTTATGTTCAATTTTC<br>R: CAAGAAAACACAAAATCCATTACTTG	$(ATTA)_5$	5'-FAM	147
SA-te-98	F: GGTGGATTTGCTGATTATTTTGT<br>R: TTGCCACTTGATCTTATCTTGCT	$(TTAA)_5$	5'-FAM	109
